# Structure of Pigment Yellow 181 dimethylsulfoxide *N*-methyl-2-pyrrolidone (1:1:1) solvate from XRPD + DFT-D

**DOI:** 10.1107/S2052520615000724

**Published:** 2015-02-01

**Authors:** Jacco van de Streek

**Affiliations:** a Institute for Inorganic and Analytical Chemistry, Frankfurt University, Max-von-Laue-Str. 7, D-60438 Frankfurt am Main, Germany; b Department of Pharmacy, University of Copenhagen, Universitetsparken 2, 2100 Copenhagen, Denmark

**Keywords:** Pigment Yellow 181, X-ray powder diffraction, dispersion-corrected density functional theory

## Abstract

With only a 2.6 Å resolution laboratory powder diffraction pattern of the θ phase of Pigment Yellow 181 (P.Y. 181) available, crystal-structure solution and Rietveld refinement proved challenging; especially when the crystal structure was shown to be a triclinic dimethylsulfoxide *N*-methyl-2-pyrrolidone (1:1:1) solvate. The crystal structure, which in principle has 28 possible degrees of freedom, was determined in three stages by a combination of simulated annealing, partial Rietveld refinement with dummy atoms replacing the solvent molecules and further simulated annealing. The θ phase not being of commercial interest, additional experiments were not economically feasible and additional dispersion-corrected density functional theory (DFT-D) calculations were employed to confirm the correctness of the crystal structure. After the correctness of the structure had been ascertained, the bond lengths and valence angles from the DFT-D minimized crystal structure were fed back into the Rietveld refinement as geometrical restraints (‘polymorph-dependent restraints’) to further improve the details of the crystal structure; the positions of the H atoms were also taken from the DFT-D calculations. The final crystal structure is a layered structure with an elaborate network of hydrogen bonds.

## Abstract 2

The relatively complex structure of a triclinic disolvate was solved from low-resolution laboratory powder diffraction data through the intermediate use of dummy atoms and the combination with quantum-mechanical calculations.

## 1. Introduction   

With hardware and software becoming increasingly more powerful, it is now possible to solve crystal structures even from relatively limited experimental diffraction data. This opens up new possibilities for crystal structure determination in the absence of high-quality data, *e.g.* when dealing with highly unstable phases or when the sample shows poor crystallinity (David *et al.*, 2005 ▶; Schmidt *et al.*, 2005 ▶). When the diffraction data are barely sufficient, they may be combined with data from alternative sources. X-ray powder diffraction (XRPD) data of limited quality, for example, can be supplemented with solid-state NMR data (see *e.g.* Bekö *et al.*, 2012 ▶) or electron diffraction data (see *e.g.* Gorelik *et al.*, 2009 ▶).

**Figure 1:**
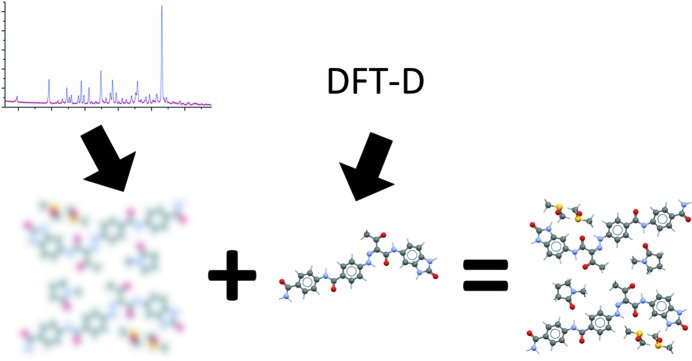
The combination of low-resolution powder diffraction and dispersion-corrected density functional theory (DFT-D): low-resolution powder diffraction data provides a poorly resolved picture of the crystal packing, which combined with the excellent bond lengths and bond angles of DFT-D calculations leads to a crystal structure with both a reliable packing and reliable molecular geometries.

Collecting more experimental data, however, relies on the assumption that the sample is still accessible. Theoretical calculations, on the other hand, can provide independent data about a system even if the sample is hard to prepare or no longer exists. Computational methods, provided that they have been carefully validated, can play an important role in the structure determination process. The combination of XRPD data with quantum-mechanical methods is especially attractive, since they complement each other’s strengths and weaknesses very well (Fig. 1 ▶). Due to peak overlap and due to reduced intensities because of the Lorentz-polarization factor in the high 2θ region of a powder pattern, the most useful information in a powder diffraction pattern is contained in the low 2θ region (below about 2.5 Å real-space resolution, for copper radiation corresponding to about 35° 2θ). This is the part of the experimental pattern that contains information about the crystal packing, but no information about individual bond lengths or valence angles. Quantum-mechanical calculations, on the other hand, are able to reproduce molecular geometries with high accuracy nowadays, and by energy-minimizing the complete crystal structure, as opposed to a single molecule in vacuum, even the subtle effects of crystal packing on molecular geometry are taken into account.

**Figure 2:**
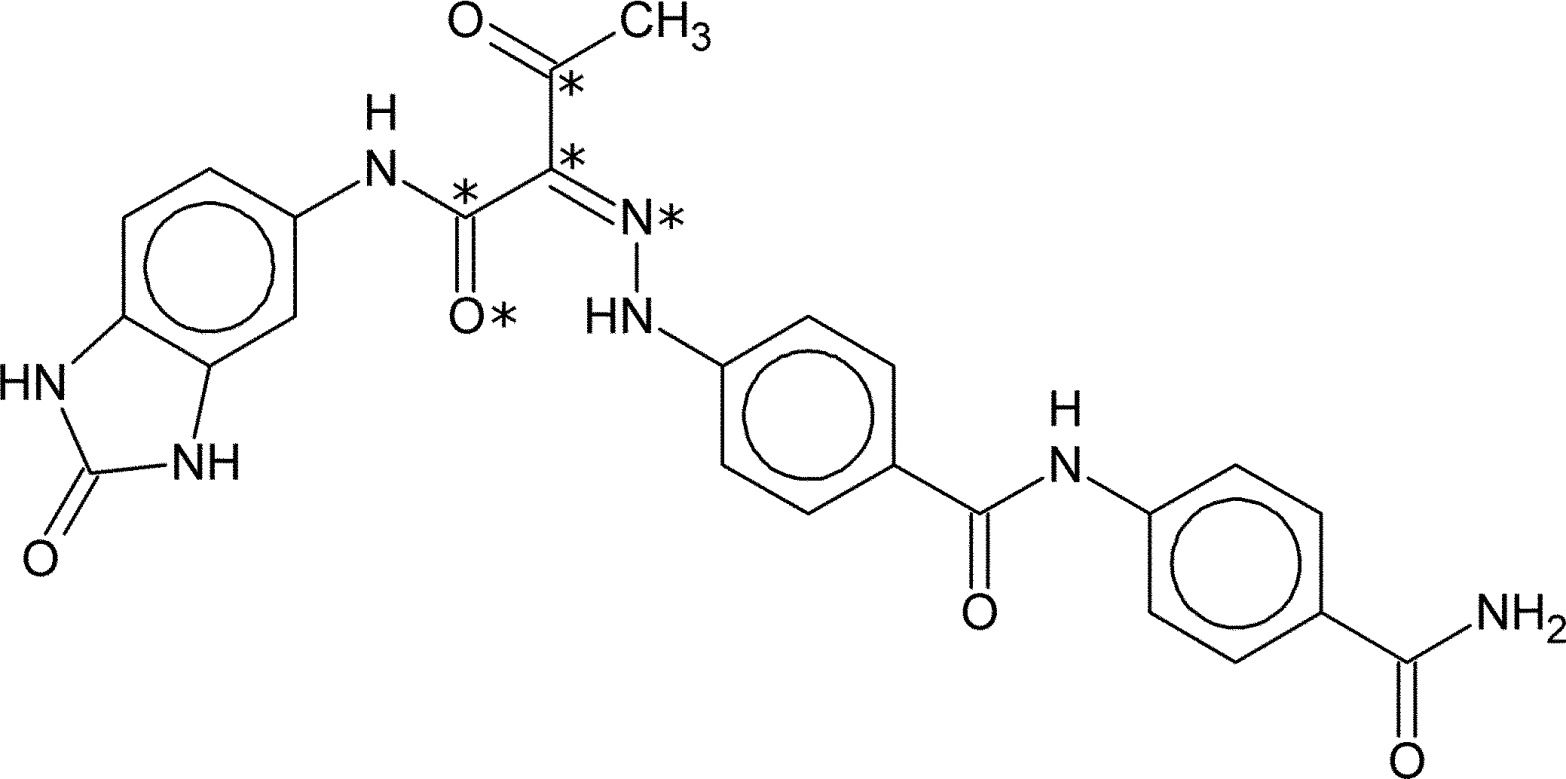
The molecular formula of Pigment Yellow 181 (P.Y. 181). Asterisks indicate the five central atoms that were restrained to be planar during Rietveld refinement.

Pigment Yellow 181 (P.Y. 181) is an organic pigment (Fig. 2 ▶), which like the majority of industrially important organic pigments is known to crystallize in multiple polymorphic forms. Extensive polymorph screening uncovered no fewer than ten crystalline phases (Hunger & Pesenacker, 1983 ▶; Schmidt & Mehltretter, 2006 ▶). P.Y. 181 is a benzimidazolone pigment, and as such it is virtually insoluble in most solvents. Single crystals can therefore not be grown, and it is common for crystal structures of industrially relevant organic pigments to be solved from powder diffraction data (see *e.g*. Van de Streek *et al.*, 2009 ▶). Of the ten polymorphic forms, only the β polymorph is of commercial interest, and it is the only polymorph for which considerable experimental data are available; we were able to report the crystal structure of the β polymorph, solved from laboratory XRPD data, earlier (Pidcock *et al.*, 2007 ▶). For the θ polymorph only limited experimental data were available, namely a single laboratory XRPD pattern with a real-space resolution of only 2.6 Å that had been measured for identification purposes as part of a polymorph screen. Since the θ polymorph is merely of academic interest, the cost of further experimental work could not be justified; we were nevertheless able to solve its crystal structure. It could be demonstrated that the structure is a solvate, and the structure-solution method that was used provides a good example of the use of dummy atoms to temporarily replace unknown solvent molecules. In the light of the poor real-space resolution of the experimental data *versus* the complexity of the final structure, even a satisfactory Rietveld refinement potentially leaves some room for uncertainty regarding the details of the crystal structure. The correctness of the crystal structure was therefore verified by means of a dispersion-corrected density functional theory (DFT-D) method that has recently been validated against 225 high-quality single-crystal crystal structures (Van de Streek & Neumann, 2010 ▶). The bond lengths and valence angles from the DFT-D calculation were then used as restraints in the Rietveld refinement. The final coordinates of the H atoms were obtained by energy-minimizing the positions of the H atoms with the non-H atoms and the unit cell kept fixed, the coordinates of the H atoms in the accompanying CIF in the supporting information therefore reflect nuclear positions rather than maxima in the electron density.

## 2. Experimental   

The θ polymorph was obtained by dissolving to completion 24 parts of P.Y. 181 in the β polymorph in a boiling mixture of 500 parts of dimethylsulfoxide (DMSO) and 500 parts of *N*-methyl-2-pyrrolidone (NMP). Slow cooling at room temperature yields the θ polymorph in powder form (Schmidt & Mehltretter, 2006 ▶).

The powder pattern was collected in transmission mode on a Stoe-Stadi-P diffractometer (Stoe & Cie, 2004 ▶) equipped with a curved Ge(111) monochromator (Cu *K*α_1_ radiation) using a linear position-sensitive detector.

### 2.1. Indexing   

21 peaks were manually selected and their positions were accurately fitted with an asymmetry-corrected full Voigt function in the program *DASH* (David *et al.*, 2006 ▶). The pattern was indexed unambiguously with a triclinic unit cell with very good figures of merit [*M*(21) = 42.0, *F*(21) = 105.3; note that this is for laboratory data] with the program *DICVOL*91 (Boultif & Louër, 1991 ▶); the Pawley fit was excellent with a χ^2^ of 1.086 for 183 reflections. The unit cell being triclinic, the space-group symmetry must be *P*1 or 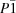. The molecule does not contain an inversion centre, and *Z*′ must therefore be an integer.

### 2.2. Crystal-structure solution   

With a unit-cell volume of 1630 Å^3^ and an estimated molecular volume for (I) of 560 Å^3^, it appeared likely that the θ polymorph, grown from a mixture of DMSO and NMP, was in fact a solvated form. Using Hofmann’s atomic volumes (Hofmann, 2002 ▶), the presence of one P.Y. 181 molecule, one DMSO molecule and one NMP molecule in the asymmetric unit seemed the most likely.

It was attempted to solve the crystal structure from the powder diffraction pattern in direct space using simulated annealing with the program *DASH* (David *et al.*, 2006 ▶). The molecular geometries were taken from experimental structures; the geometry of the P.Y. 181 molecule was taken from the crystal structure of the β polymorph (Pidcock *et al.*, 2007 ▶). All bond lengths and valence angles are fixed during the structure-solution process, as are all torsion angles around double bonds and those within rings. For a P.Y. 181 molecule, this leaves ten possible flexible torsion angles, and adding the translational and rotational degrees of freedom of one P.Y. 181, one DMSO and one NMP molecule yields a total of 28 degrees of freedom. Fortunately, due to the conjugated nature of the double bonds and the extensive possibilities for intramolecular hydrogen bonds, most of the flexible torsion angles in the P.Y. 181 molecule can be restricted to narrow ranges or can even be fixed during the simulated annealing, as described earlier (Pidcock *et al.*, 2007 ▶). Because of the large number of degrees of freedom, the number of Monte-Carlo moves per simulated annealing run was increased from its default value of 10 000 000 moves to 100 000 000 moves.

Analysis of the first ten simulated annealing solutions showed P.Y. 181 molecules that had started to form plausible networks of hydrogen bonds, with no short contacts. Between the P.Y. 181 molecules two large gaps could be identified, which were occupied by the solvent molecules. Furthermore, the only two hydrogen-bond donors of the P.Y. 181 molecule that did not participate in the hydrogen-bonded network each pointed directly into one of the two gaps, and indeed both DMSO and NMP contain an excellent hydrogen-bond acceptor and no donor. The N—H⋯O=C distances between the P.Y. 181 molecules, however, were unusually large: N⋯O distances of around 3.0 Å were measured, whereas distances of around 2.8 Å would be expected. Moreover, the orientations of the solvent molecules appeared to be random, the distribution of the DMSO and NMP molecules over the two gaps did not show any pattern either and in none of the solutions had either of the solvent molecules formed a plausible hydrogen bond to the P.Y. 181 molecule. As at this stage the exact nature of the solvent molecules was still uncertain – especially because the possibility of disorder could not be ruled out – and in view of the large number of degrees of freedom, it was first attempted to see if it was possible to determine the positions of the P.Y. 181 molecules with greater accuracy, filling in the gaps later.

**Figure 3:**
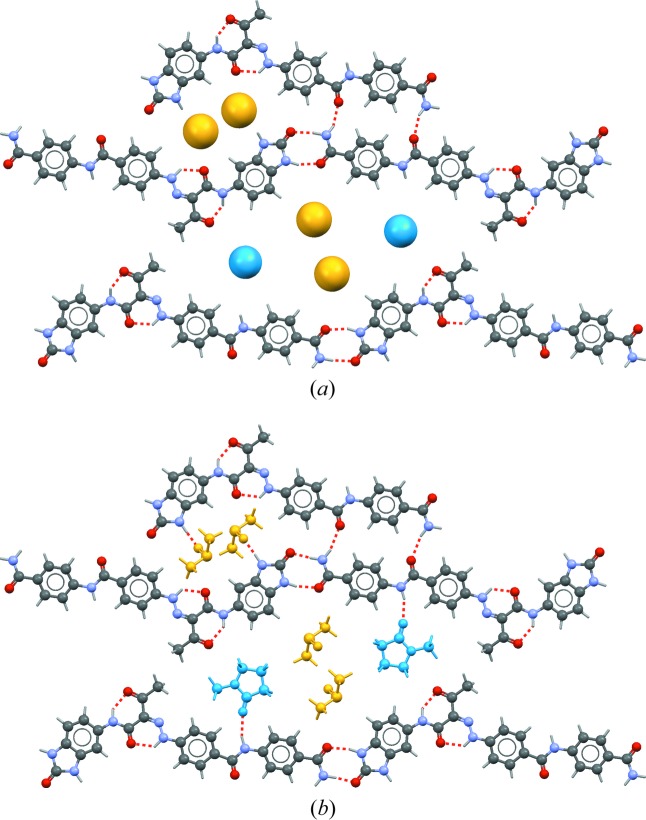
(*a*) Result of the partial Rietveld refinement with two dummy atoms to determine the exact location of the P.Y. 181 molecules; several unsatisfied N—H hydrogen-bond donors can be identified. A yellow dummy atom represents the equivalent of 41 electrons, a blue dummy atom the equivalent of 50 electrons. (*b*) Result of the final Rietveld refinement after the DMSO (yellow) and the NMP (blue) molecules had been located successfully; the N—H hydrogen-bond donors are now satisfied by the C=O and S=O groups of the solvent molecules. Hydrogen bonds are shown as red dashed lines.

To this end, the best solution found thus far was taken and its solvent molecules were removed. In their stead, two dummy atoms were inserted whose positions, occupancies and isotropic displacement parameters were allowed to refine freely; a similar approach had earlier been used successfully by Dinnebier *et al.* (1997 ▶). Restraints were applied to keep the geometry of the P.Y. 181 molecule chemically reasonable, but its position and orientation were allowed to refine. With this model, a Rietveld refinement was carried out with the program *TOPAS-Academic* (Coelho, 2007 ▶). The Rietveld refinement remained stable and converged quickly and smoothly, after which the P.Y. 181 molecules formed an excellent network of hydrogen bonds (Fig. 3 ▶ *a*) with N⋯O distances of around 2.8 Å as expected. The occupancies of the two dummy atoms had refined to values corresponding to 41 and 50 electrons, whereas 42 and 54 would have been expected for DMSO and NMP, respectively. This gave great confidence that the correct position and orientation of the P.Y. 181 molecule had been found, and that the missing solvent molecules should indeed be one molecule of DMSO and one molecule of NMP. For completeness, we mention that the *B*_iso_ values of the two dummy atoms had refined to 65 Å^2^ and 57 Å^2^ for ‘DMSO’ and ‘NMP’ respectively.

Therefore, new simulated annealing runs were started with the program *DASH*, this time with the geometry, position and orientation of the P.Y. 181 molecule fixed at the values obtained from the Rietveld refinement. The positions and orientations of the DMSO and the NMP molecules were easily found this way (Fig. 3 ▶ *b*). As can be seen from Fig. 3 ▶, the number of electrons in the dummy atoms correctly predicted the respective locations of the DMSO and the NMP molecules. The excellent agreement between the number of electrons in the dummy atoms and the true solvent molecules should, however, be considered fortuitous and in general the identification based on the dummy atoms may be more troublesome. Both solvent molecules accept hydrogen bonds, as was predicted from the two unsatisfied donors in the hydrogen-bonding framework formed by the P.Y. 181 molecules.

### 2.3. DFT-D energy-minimization   

The real-space resolution of the experimental powder diffraction pattern being only 2.6 Å, it could be argued that it is questionable if such a complicated crystal structure, with three molecules in the asymmetric unit, can be reliably solved from it. Since only the β polymorph is of commercial interest, the crystal structure of the θ polymorph is merely of academic interest and no additional experimental data are available. Recently, however, a paper was published that validates a dispersion-corrected density functional theory (DFT-D) method that reproduces crystal structures of molecular compounds well enough to distinguish between correct and incorrect experimental organic crystal structures (Van de Streek & Neumann, 2010 ▶). The crystal structure of P.Y. 181 dimethylsulfoxide *N*-methyl-2-pyrrolidone (1:1:1) solvate was energy-minimized with *CASTEP* (Clark *et al.*, 2005 ▶) using the Perdew–Burke–Ernzerhof (PBE) functional with the Grimme (2006 ▶) dispersion correction and a cut-off energy of 520 eV. Such a DFT-D calculation provides an independent check of the correctness of the crystal structure. The relevant quantity is the r.m.s. Cartesian displacement upon energy-minimization with the unit cell free, where H atoms are excluded from the comparison. For the validation set of 225 molecular single-crystal structures, the average r.m.s. Cartesian displacement was 0.084 Å. For correct crystal structures, r.m.s. Cartesian displacements of up to 0.25 Å were found whereas incorrect crystal structures gave values over 0.30 Å. For the P.Y. 181 crystal structure, a value of 0.17 Å was found, which proves beyond reasonable doubt that the crystal structure is correct.

### 2.4. Final Rietveld refinement   

*TOPAS-Academic* (Coelho, 2007 ▶) was used for Rietveld refinement of the final crystal structure. For the Rietveld refinement, chemical restraints were added for all bond lengths and valence angles based on the DFT-D energy-minimized structure. The crystal structure is energy-minimized as a whole and the energy-minimized bond lengths and valence angles therefore include the packing effects for this particular polymorph; we call these ‘polymorph-dependent restraints’. Three planarity restraints were added for the three aromatic ring systems in the P.Y. 181 molecule, a fourth planarity restraint was added for the five central atoms of the molecule marked with an asterisk in Fig. 2 ▶. The definitions of the planarity restraints were also guided by the energy-minimized structure. The entire benzimidazolone group was treated as one aromatic system. A planarity restraint was also added for all non-H atoms in the NMP molecule except for the central CH_2_ group. However, although the central CH_2_ group could in principle be bent out of the plane, CSD searches show that this is seldom the case. Each of the three molecules was assigned a separate isotropic displacement parameter *B*_iso_. The Rietveld refinement suffered from several problems connected with parameters being highly correlated. A sensible set of parameters was obtained by fixing the *B*_iso_ values at 3.0 Å^2^ for the P.Y. 181 and the NMP molecules and at 5.0 Å^2^ for the DMSO molecule, which gave an excellent fit to the experimental data without any need for a preferred orientation correction.

**Figure 4:**
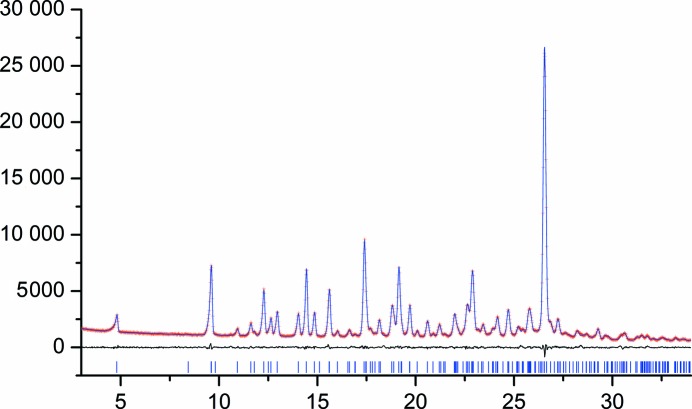
Fit of the calculated to the experimental PXRD pattern after Rietveld refinement (*x*-axis: °2θ; *y*-axis: counts). Calculated (blue), observed (red) and difference (black) profiles are shown. Tick marks are shown at the bottom of the profile.

The final figures of merit of the Rietveld refinement are χ^2^ = 1.43, *R*_wp_ = 0.0317 and *R*_p_ = 0.0237 (without background subtraction), 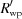 = 0.0616 and 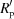 = 0.0540 (after background subtraction). Fig. 4 ▶ shows the fit after the Rietveld refinement. The crystallographic parameters of the final crystal structure are listed in Table 1 ▶.

The molecular geometry was checked using *Mogul* (Bruno *et al.*, 2004 ▶) to compare all bond lengths and valence angles against distributions from single-crystal data. The relevant measure is the absolute value of the *z*-score, which measures how many standard deviations each value in the P.Y. 181 crystal structure differs from the mean of the distribution from the single-crystal data. For the P.Y. 181 structure, the largest value is 2.8 for one of the bond lengths and 2.5 for one of the bond angles; in other words, none of the bond lengths or bond angles in the final structure is more than 2.8 standard deviations away from its mean single-crystal value.

The overall *B*_iso_ values of the DMSO molecule were persistently higher than the overall *B*_iso_ values of the P.Y. 181 and the NMP molecules, which could indicate disorder. A Rietveld refinement with a disordered model was attempted, the occupancies of the S atoms refined to 0.86 and 0.14. There was no significant improvement to the agreement parameters of the Rietveld refinement, and the real-space resolution of the powder diffraction pattern is too low to allow definitive statements about the disorder of the DMSO group. Attempts to resolve the disorder by means of DFT-D calculations were inconclusive. We conclude that the DMSO molecule is probably disordered, with one distinct major occupancy. The CIF in the supporting information only reflects this major occupancy.

## 3. Discussion and conclusion   

In the case of θ-P.Y. 181, the DFT-D calculations were primarily needed because of the scarcity of the experimental data; in general, however, checking crystal structures from powder diffraction data against information available from independent sources is always recommended, even in cases where experimental data of acceptable resolution are available. Ideally, each crystal structure determined from powder diffraction data should be energy-minimized with dispersion-corrected density functional theory to confirm the correctness of the structure; the bond lengths and valence angles of the energy-minimized crystal structure should then be fed back into the Rietveld refinement as ‘polymorph-dependent’ restraints.

Although our diffraction data have a limited real-space resolution, the data are by no means of poor or low quality: there are 30 000 counts in the highest peak, the zero-point error is less than 0.04° 2θ, the full width at half maximum is acceptable (certainly for an organic pigment), the background is flat and there is no preferred orientation.

Further analysis into the failure of the initial simulated annealing runs to locate the three molecules correctly showed the failure to be caused by the difference in the molecular geometries of the P.Y. 181 molecule (mainly differences in valence angles) in the θ polymorph and the β polymorph (from which the initial geometry was taken).

With hindsight, the θ polymorph was determined to be a solvate, with the two solvent molecules accounting for no less than 27% of the electron density in the crystal structure. We were able to solve the structure by alternating structure solution and partial refinement; we note that this is common practice in structure determinations from single-crystal data. For completeness, we mention that mathematically more elegant methods exist to cope with the presence of unknown solvent molecules, namely the use of maximum likelihood methods (for an example of an application to powder diffraction data, see Markvardsen *et al.*, 2002 ▶, and the *SQUEEZE* algorithm, van der Sluis & Spek, 1990 ▶). Whether these methods would have worked with the given limited data quality and the large fraction of electron density missing in this particular case was not investigated.

**Figure 5:**
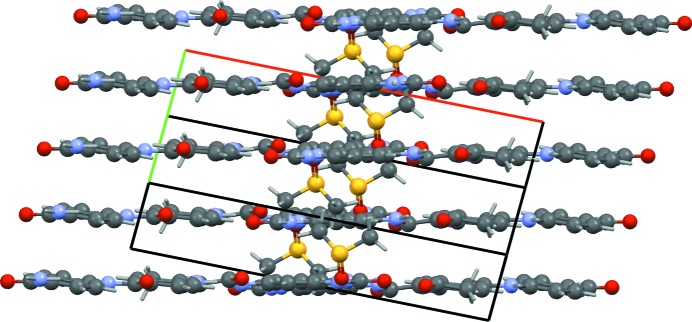
Side view of the layers shown in Fig. 3 ▶. The P.Y. 181 and the NMP molecules lie in one plane.

The final crystal structure contains no unreasonable short contacts. The packing shows excellent hydrogen bonds, with all hydrogen-bond donors and acceptors satisfied with good geometries (Fig. 3 ▶). The P.Y. 181 molecules are planar and pack in layers consisting of parallel infinite one-dimensional chains of hydrogen-bonded molecules (Fig. 5 ▶). The molecular geometry, refined with restraints, agrees with existing single-crystal data. The packing and the molecular geometry are confirmed by dispersion-corrected density functional theory (DFT-D) calculations.

Using Hofmann’s volumes for the DMSO and the NMP molecules, the molecular volume of a P.Y. molecule in the θ polymorph is 1628.78/2 − (94.81 + 138.26) ≃ 581 Å^3^. The molecular volume of the β polymorph being 2246.16/4 ≃ 562 Å^3^, it is clear that the θ polymorph is less dense than the β polymorph.

## Supplementary Material

Crystal structure: contains datablock(s) I. DOI: 10.1107/S2052520615000724/zb5045sup1.cif

Rietveld powder data: contains datablock(s) I. DOI: 10.1107/S2052520615000724/zb5045Isup2.rtv

Crystal structure energy-minimised with PBE. DOI: 10.1107/S2052520615000724/zb5045sup3.txt

CCDC reference: 1043397

## Figures and Tables

**Table 1:** Structural parameters

*a* ()	18.4436(11)
*b* ()	8.4454(4)
*c* ()	10.9223(6)
()	106.255(2)
()	90.268(3)
()	94.002(3)
*V* (^3^)	1628.78(16)
*Z*/*Z*	2/1
Space group	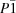
Chemical formula	C_25_H_21_N_7_O_5_, C_2_H_6_OS, C_5_H_9_NO

## References

[bb2] Bekö, S. L., Urmann, D., Lakatos, A., Glaubitz, C. & Schmidt, M. U. (2012). *Acta Cryst.* C**68**, o144–o148. 10.1107/S0108270112005252 22382550

[bb3] Boultif, A. & Louër, D. (1991). *J. Appl. Cryst.* **24**, 987–993.

[bb4] Bruno, I. J., Cole, J. C., Kessler, M., Luo, J., Motherwell, W. D. S., Purkis, L. H., Smith, B. R., Taylor, R., Cooper, R. I., Harris, S. E. & Orpen, A. G. (2004). *J. Chem. Inf. Comput. Sci.* **44**, 2133–2144. 10.1021/ci049780b 15554684

[bb5] Clark, S. J., Segall, M. D., Pickard, C. J., Hasnip, P. J., Probert, M. J., Refson, K. & Payne, M. C. (2005). *Z. Kristallogr.* **220**, 567–570.

[bb6] Coelho, A. A. (2007). *TOPAS-Academic*, Version 4.1. Bruker AXS Inc., Karlsruhe, Germany.

[bb7] David, W. I. F., Shankland, K., Pulham, C. R., Blagden, N., Davey, R. J. & Song, M. (2005). *Angew. Chem. Int. Ed.* **44**, 2–6. 10.1002/anie.200501146 16222650

[bb8] David, W. I. F., Shankland, K., Van de Streek, J., Pidcock, E., Motherwell, W. D. S. & Cole, J. C. (2006). *J. Appl. Cryst.* **39**, 910–915.

[bb9] Dinnebier, R. E., Pink, M., Sieler, J. & Stephens, P. W. (1997). *Inorg. Chem.* **36**, 3398–3401. 10.1021/ic961385i 11670012

[bb10] Gorelik, T., Schmidt, M. U., Brüning, J., Bekö, S. & Kolb, U. (2009). *Cryst. Growth Des.* **9**, 3898–3903.

[bb11] Grimme, S. (2006). *J. Comput. Chem.* **27**, 1787–1799. 10.1002/jcc.20495 16955487

[bb12] Hofmann, D. W. M. (2002). *Acta Cryst.* B**58**, 489–493. 10.1107/s0108768101021814 12037338

[bb13] Hunger, K. & Pesenacker, M. (1983). Patent EP 0 010 273 B1.

[bb14] Macrae, C. F., Bruno, I. J., Chisholm, J. A., Edgington, P. R., McCabe, P., Pidcock, E., Rodriguez-Monge, L., Taylor, R., Van de Streek, J. & Wood, P. A. (2008). *J. Appl. Cryst.* **41**, 466–470.

[bb15] Markvardsen, A. J., David, W. I. F. & Shankland, K. (2002). *Acta Cryst.* A**58**, 316–326. 10.1107/s010876730200510x 12089454

[bb16] Pidcock, E., Van de Streek, J. & Schmidt, M. U. (2007). *Z. Kristallogr.* pp. 713–717.

[bb17] Schmidt, M. U., Ermrich, M. & Dinnebier, R. E. (2005). *Acta Cryst.* B**61**, 37–45. 10.1107/S010876810402693X 15659856

[bb18] Schmidt, M. U. & Mehltretter, G. (2006). Patent WO 2006005408.

[bb19] Sluis, P. van der & Spek, A. L. (1990). *Acta Cryst.* A**46**, 194–201.

[bb20] Stoe & Cie (2004). *WinXPOW.* Stoe & Cie, Darmstadt, Germany.

[bb22] Van de Streek, J., Brüning, J., Ivashevskaya, S. N., Ermrich, M., Paulus, E. F., Bolte, M. & Schmidt, M. U. (2009). *Acta Cryst.* B**65**, 200–211. 10.1107/S0108768108041529 19299876

[bb21] Van de Streek & Neumann, M. A. (2010). *Acta Cryst.* B**66**, 544–558. 10.1107/S0108768110031873 PMC2940256 20841921

